# Deep Eutectic Solvents for Efficient and Selective Extraction of α-Glucosidase Inhibitors from Waste Seeds of Refined Betel Nuts

**DOI:** 10.3390/foods13071070

**Published:** 2024-03-30

**Authors:** Jin Liu, Li Ma, Senwen Deng, Xinzhi Chen, Qi Li, Aiqing Xu, Ting Tong, Shuhua Tan, Mingkang Wang, Jiangtao Cai, Haihua Wang

**Affiliations:** 1 Hunan Key Laboratory of Economic Crops Genetic Improvement and Integrated Utilization, School of Life and Health Sciences, Hunan Academy of Binglang Science, Hunan University of Science and Technology, Xiangtan 411201, China; liujin1601243357@mail.hnust.edu.cn (J.L.); cxzw@mail.hnust.edu.cn (X.C.); aqxu@hnust.edu.cn (A.X.); tongting0430@hnust.edu.cn (T.T.); shtan@hnust.edu.cn (S.T.);; 2 National Engineering Research Center of Oiltea Camellia, Research Institute of Oiltea Camellia, Hunan Academy of Forestry, Shao Shan South Road, No. 658, Changsha 410004, China; supermarry1@hnlky.cn; 3 Hunan Lerkam Biology Corp., Ltd. No. 12, Rongxin Road, Ecological Industrial Park, Chenguan Town, Yueyang 414009, China; 4 Hunan Vocational Institute of Safety Technology, Wang Jiali North Road, No. 66, Changsha 410151, China

**Keywords:** deep eutectic solvent, extraction, α-glucosidase inhibition, refined betel nut

## Abstract

During the production process of refined betel nuts in China, a large amount of processing by-product, betel nut waste seeds, is generated. Betel nut waste seeds are rich in bioactive elements, but they have not been effectively utilized yet. In this study, an ultrasonic-assisted deep eutectic solvent method (DES) was used to selectively extract α-glucosidase inhibitors from waste seeds. Compared with traditional extraction solvents such as water and ethanol, the extraction efficiency of specific DESs is higher, and the content of alkaloids in the extracts is lower. However, it should be noted that some pure DESs exhibit inhibitory activity towards α-glucosidase. DESs, based on choline chloride/urea, were selected due to the high extraction efficiency of α-glucosidase inhibitors and their low alkaloid content as well as low inhibitory activity. The optimal extraction conditions were determined using single-factor experiments as follows: 30% (*v*/*v*) water content, a choline chloride/urea ratio of 5:3, a solid–liquid ratio of 1:10, extraction temperature of 40 °C, and a duration of 30 min. Through recovery experiments, it was found that the DES can be reused four times under these conditions, maintaining an inhibition rate comparable to alcohol extraction methods. The IC_50_ value of the extract was measured at 0.0066 mg/mL, superior to acarbose. In summary, this research has successfully developed an efficient and selective method for extracting α-glucosidase inhibitors from betel nut waste seeds, thereby presenting a promising avenue for future applications.

## 1. Introduction

Betel nut, derived from the fruit of *Areca catechu* L., has received global interest for its wide-ranging medicinal and dietary applications [1]. In China, betel nut is primarily cultivated in provinces such as Hainan and Yunnan. Specifically, in Hainan Province, the cultivation and harvest areas for betel nut encompass 0.15 and 0.08 million hectares, respectively. Moreover, the province’s production of fresh and dried betel nut fruits has reached 600,000 and 272,200 tons, respectively [2,3]. Betel nuts can be consumed in three primary forms: chewing fresh betel nuts, dried kernels, and dried betel nut pericarp [4,5]. In Hainan Province, over 95% of the harvested fruits are sent to Hunan Province and processed into refined betel nuts, locally referred to as “Binglang”, after its intricate processing steps, including boiling, flavoring, denucleation, brining, and drying [3]. The denucleation process involves slicing the betel nut in half to remove the seeds. Annually, the betel nut industry generates approximately 35,000 tons of waste seeds [6]. While a fraction of these seeds are utilized for bioactive compound extraction, the majority are discarded or incinerated, leading to ineffective resource use and environmental pollution [7].

The main components of betel nut seeds are 31.1% polyphenols, 18.7% polysaccharides, 14.0% fats, 10.8% crude fiber, 9.9% moisture, 3.0% ash, and 0.5% alkaloids [8]. The specific content of these substances varies with the processing technology. These components offer several beneficial effects, including digestion promotion, antioxidant and anti-inflammatory properties, parasite resistance, and antimicrobial activity [1,9,10,11]. The phenolic substances are mainly flavonoids, including 10% catechins, 2.5% epicatechins, and 12% leucoanthocyanins [6]. All of these substances have inhibitory effects on α-glucosidase. Among them, catechins and epicatechins have higher inhibitory activity than acarbose [12,13]. Limited studies have explored the extraction of bioactive substances from betel nut waste seeds using traditional methods [6]. These extraction techniques often rely on organic solvents. These solvents suffer from drawbacks such as low extraction efficiency, high levels of impurities, stringent extraction conditions, significant costs, and environmental harm. As a result, the commercial and practical appeal of the extracted product is diminished [14]. Deep eutectic solvents (DESs), characterized by their affordability, non-toxicity, eco-friendliness, straightforward preparation, and biodegradability, present numerous advantages over traditional solvents. Consequently, they hold considerable promise for extracting plant bioactive components [15]. By fine-tuning the hydrogen bonding network, DESs can be tailored and optimized for selective extraction processes [16]. Presently, DESs are extensively applied in the extraction of a variety of compounds, including flavonoids, lignins, phenols, alkaloids, polysaccharides, and essential oils [15]. Research on optimizing the extraction of bioactive components from betel nut waste seeds with DESs remains scarce. Moreover, the specific compositions and optimal conditions for such DES extractions are still to be determined [17].

This study endeavors to scrutinize the extraction process of α-glucosidase inhibitors from betel nut waste seeds utilizing DES. The primary aim is to optimize the extraction process of bioactive compounds from discarded betel nut seeds while diminishing the presence of impurities such as alkaloids in the extracted products.

## 2. Materials and Methods

### 2.1. Reagents and Materials

4-Nitrophenyl-*α*-D-glucopyranoside (*p*-NPG) and α-glucosidase were provided by Sigma-Aldrich Chemical Co., Ltd. (Shanghai, China). Organic solvents and the Folin–Ciocalteu reagent were purchased from Sinopharm Chemical Reagent Co., Ltd. (Shanghai, China). Arecoline hydrobromide, rutin trihydrate, and gallic acid were purchased from Aladdin Biochemical Technology Co., Ltd. (Shanghai, China). Choline chloride (98%), betaine (98%), L-proline (99%), glucose (99%), lactic acid (88%), xylitol (99%), malic acid (98%), urea (99%), citric acid (99.5%), glycerol (99%), 1,4-butanediol (98%), ethylene glycol (99.5%), and AB-8 macroporous adsorption resin were purchased from Macklin Biochemical Co., Ltd. (Shanghai, China). All these reagents were of analytical grade.

### 2.2. Preparation of DES

A total of thirteen types of DESs were synthesized, following the procedure documented in prior work [18]. Table 1 displays the hydrogen bond donors and acceptors utilized in the preparation of these DESs. The components were mixed in the recommended molar ratios. The resulting mixtures were stirred using a magnetic stirring apparatus at 80 °C until they became a viscous, stable, and transparent liquid (DES). A minor quantity of water (6.25%, *w*/*w*) was introduced during the preparation of ChCl-Urea and Bet-CA to diminish the system’s viscosity. Moreover, the lactic acid utilized harbored 12% aqueous impurities. Consequently, the prepared DESs, including ChCl-Urea, Bet-CA, ChCl-LA, Bet-LA, and Pro-LA, were subjected to rotary evaporation at 90 °C until the water was removed as much as possible. After rotary evaporation, mass analysis revealed that the water content of DESs decreased to 4.53%, 4.65%, 6.57%, 6.73%, and 6.80% (*w*/*w*), respectively.

### 2.3. Extraction of Phytochemical Constituents from Waste Seeds

Processed betel nut waste seeds were provided by Hunan New Sensation Supply Chain Management Co., Ltd. (Yueyang, China). The standard processing of betel nuts encompasses a series of steps as follows: blanching, drying, cooking, steam explosion, fermentation, fragrance enhancement, compressing, and top coating [3]. Following these procedures, the betel nuts were bisected, and the seeds were gathered. These waste seeds were dried to a constant weight at a temperature of 60 °C, subsequently pulverized into a fine powder using a micromill and sifted through a 40-mesh sieve (0.425 mm) to obtain a uniform-size powder. To diminish the viscosity of the DES, 30% (*v*/*v*) water addition was implemented in all DESs [18]. For the extraction process, 1.0 g of the resulting powder was mixed with 5 mL of the extraction solvent. Ultrasonic treatment of 400 W (Scientz-250C, Ningbo Scientz Biotechnology Co., Ltd., China (Ningbo, China)) at a temperature of 30 °C was administered for 30 min. The extract was first subjected to centrifugation at 10,000× *g* for 10 min for solid–liquid separation. The supernatant obtained was stored at 4 °C and then analyzed as soon as possible.

### 2.4. Total Phenolic Content, Total Flavonoid Content and Total Alkaloid Content Determinations

The total phenolic content (TPC) was quantified utilizing the ferrous tartrate method, as described in a previous study [19]. Gallic acid was used as the reference standard. A volume of 0.3 mL of the extract derived from betel nut waste seeds was transferred into a 5 mL volumetric flask. Distilled water was added to bring the volume up to 1 mL, followed by the addition of 1 mL of the ferrous tartrate solution. The flask was subsequently filled up to the calibration line with a phosphate-buffered solution at pH 7.5. The contents were thoroughly combined and allowed to rest undisturbed for 15 min. The absorbance value was then measured at 540 nm. The TPC values were represented as milligrams of gallic acid equivalents per gram of the dried samples (mg GAE/g DW).

The determination of the total flavonoid content (TFC) was performed using the aluminum chloride colorimetric method, as described by reference [20], with rutin serving as the standard. For sample preparation, 100 μL of the solution was mixed with 40 μL of the 0.5 M NaNO_2_ solution. After a rest period of 5 min, 40 μL of the 0.3 M AlCl_3_ solution was added and left to stand for an additional 6 min. Subsequently, 400 μL of 0.5 M of NaOH solution was added, and the mixture was vigorously agitated for 30 s. The absorbance was measured at 510 nm. The results were expressed as milligrams of rutin equivalents per gram of the dried samples (mg RE/g DW).

The total alkaloid content (TAC) was determined following the method described, with arecoline as the standard [21]. An appropriate amount of betel nut waste seed extract was deposited into a separating funnel. Subsequently, 4 mL of the acid–sodium acetate buffer at pH 5.0 and 2 mL of the bromocresol green solution were cautiously added. The mixture was gently agitated to ensure thorough mixing. The extraction process was conducted in three stages, with sequential additions of 2, 3, and 5 mL of chloroform. Each extraction was accompanied by a 2 min shaking period, followed by a 30 min settling period. The chloroform layer was then collected and transferred to a dry volumetric flask containing 0.2 g of anhydrous sodium sulfate. After shaking and a 30 min settling period, 5 mL of the supernatant was transferred to a conical flask. Subsequently, 1 mL of 0.01M potassium hydroxide in anhydrous ethanol was added, and the mixture was thoroughly shaken before the absorbance was measured at a wavelength of 618 nm. A blank control using distilled water was employed. The values were expressed as milligrams of arecoline equivalents per gram of the dried samples (mg AE/g DW).

### 2.5. Evaluation of α-Glucosidase Inhibitory Activity

The α-glucosidase inhibitory activity (α-GIA) of the extracts was evaluated utilizing the method described in the referenced study [22]. The reaction system consisted of 100 μL of a 100 mM phosphate buffer (pH 7.0), 80 μL of 20 mM *p*-NPG, 2 μL of the inhibitor, and 20 μL of the enzyme. Acarbose (0.01mg/mL) was used as a positive control. This mixture was then incubated in a water bath at 37 °C for 20 min. The reaction was halted by the addition of 400 μL of a 0.2 M Na_2_CO_3_ solution, and the change in absorbance was measured at 400 nm. Compared with the absorbance value of the blank group, the inhibition rate of the enzyme could be calculated. The half inhibitory concentration value (IC_50_) was determined by plotting inhibition–concentration curves using non-linear regression analysis.

### 2.6. Optimization of Extraction Conditions

To determine the optimal range of extraction conditions, single-factor experiments were conducted. Tested variables encompassed the water content (10%, 20%, 30%, 40%, 50%, 60%, *v*/*v*), the molar ratio of choline chloride to urea (5:1, 5:2, 5:3, 5:4, 5:5), the solid–liquid ratio (1:5, 1:10, 1:15, 1:20, 1:25, 1:30), temperature (20 °C, 30 °C, 40 °C, 50 °C, 60 °C), and time (10 min, 20 min, 30 min, 40 min, 50 min). The α-GIA and TAC of the extracts were measured.

### 2.7. Recovery of DESs and Inhibitors

This separation was conducted via a glass column (10 mm × 500 mm) wet-packed with 10 g of AB-8 macroporous resin (dry weight), with the BV at 20 mL. The extraction solution was passed through the column at a flow rate of 30 mL per hour. Upon reaching adsorptive equilibrium, the column was first rinsed with 200 mL of deionized water. Subsequently, it was eluted with a 60% ethanol solution (*v*/*v*) at a flow rate of 30 mL per hour. The ethanolic eluents were then concentrated using vacuum rotary evaporation and freeze-drying. The water eluents were concentrated through vacuum rotary evaporation (90 °C, 30 min) for the recovery of the DES, and the obtained DESs were reused for extraction until the inhibition rate of the extracts was lower than that of the ethanolic extracts [23,24].

### 2.8. Statistical Analysis

All experiments were replicated thrice, and the results were expressed as means ± standard deviations. The data were subjected to statistical and multivariate analyses using IBM SPSS Statistics version 17.0 software (IBM SPSS Inc., Armonk, NY, USA). A *p*-value less than 0.05 was deemed statistically significant.

## 3. Results and Discussion

### 3.1. Influence of Extraction Solvent

**Figure 1:**
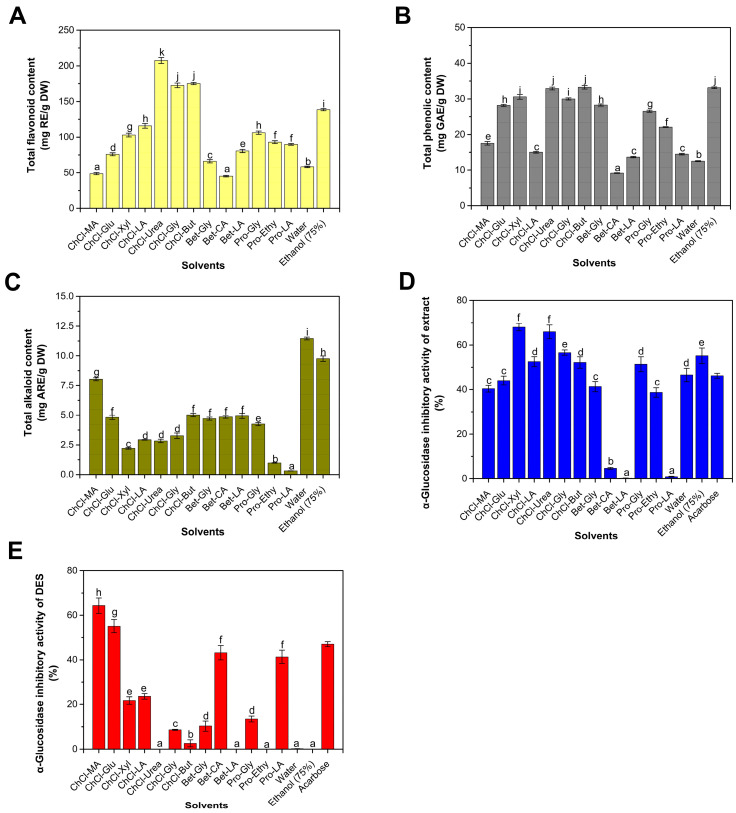
Total flavonoid content (**A**), total phenolic content (**B**), total alkaloid content (**C**) and α-glucosidase inhibitory activity (**D**) of the betel nut waste seed extracts. α-glucosidase inhibitory activity of pure DESs (**E**). Different lowercase letters indicated statistically significant differences.

This study, along with other research findings, demonstrates that waste seeds from refined betel nuts contain abundant bioactive compounds, including total flavonoids, total polyphenols, and alkaloids, with a particularly high concentration of flavonoids [6,17]. The previous literature has shown that DESs used in this study have a good extraction effect on plant-derived flavonoids and other polyphenols [17,18]. The quantity of these substances is significantly influenced by the type of extraction solvent (Figure 1).

#### 3.1.1. Influence of Extraction Solvent on TPC and TFC

The aqueous extraction method resulted in an extract with a flavonoid content of 58.34 mg/g and a phenolic content of 8.94 mg/g (Figure 1A,B). The alcohol extraction method produced an extract with a flavonoid content of 138.44 mg/g and a phenolic content of 33.12 mg/g. Among the tested DESs, the flavonoid content ranged from 45.12 to 207.48 mg/g, while the phenolic content ranged from 9.16 to 33.25 mg/g. The highest flavonoid extraction rate was achieved using a DES based on choline chloride and urea, which resulted in a 49.87% increase in the extraction yield compared to the alcohol extraction. The highest phenolic extraction rate was attained with a DES based on choline chloride and 1,4-butanediol, exhibiting a comparable efficiency to that of alcohol extraction. The enhanced dissolution capacity of flavonoids can be ascribed to the hydrogen bonds formed between the solvent and extracted flavonoids [25].

#### 3.1.2. Influence of Extraction Solvent on TAC

The aqueous extract has the highest concentration of alkaloid at 11.46 mg/g, followed by the ethanol extract at 9.75 mg/g (Figure 1C). The DES used in this study exhibits a relatively low extraction rate of alkaloids. Compared to traditional water extraction methods, the extraction rate of alkaloids was reduced by over 21.42% using DES. Specifically, the extraction rates of alkaloids are the lowest for DESs based on L-proline/ethylene glycol and L-proline/lactic acid, measuring 0.98 mg/g and 0.32 mg/g, respectively. This indicates that the DES investigated in this study had a lower affinity for alkaloids compared to water and alcohol. The alkaloid content in this study was significantly lower than that reported in the literature [6,17]. Previous studies in the literature employed fresh betel nut fruits, which were directly opened to obtain the seeds, whereas this study utilized the waste seeds of processed betel nut. The processing of refined betel nuts involves steps such as steam explosion, enzymatic hydrolysis, and cooking [3]. In particular, steam explosion, characterized by high temperatures and high pressures, may damage some heat-sensitive substances [26]. The bound alkaloids or polyphenols were gradually converted into the free form and migrated from the betel nut to the solution, resulting in a substantial decrease in their content [3]. The long-term use of arecoline on diabetic animal models has shown clear cytotoxicity, causing hydropic degeneration and fatty vacuoles in the liver, suggesting that the use of processed waste seeds is more suitable for the extraction of bioactive compounds compared to fresh seeds [27]. DESs might have complex multiple interactions with target products like flavonoids, leading to selective production, which has clear benefits for the long-term use of extracts [25].

#### 3.1.3. Inhibitory Activity of Extract and Pure DES

The inhibition rate of the water extract is 46.56%, and the inhibition rate of the alcohol extract is 55.15% (Figure 1D), which is higher than 46.2% of the acarbose control (0.01 mg/mL), indicating that betel nut waste seeds contain α-glucosidase inhibitors. Among the 13 DESs tested, the inhibition rates of α-glucosidase range from 10.04% to 68.04%. Considering previous reports regarding the inhibitory effect of DESs on α-glucosidase [28], we conducted measurements of the inhibitory activity of pure DESs. We found that some DESs exhibit clear inhibitory activity on α-glucosidase, with no activation effects observed (Figure 1E). Among them, the DES based on choline chloride and malic acid exhibited the highest inhibitory activity, with an inhibition rate of 64.30%. This was followed by DESs based on choline chloride and glucose, betaine and citric acid, and proline and lactic acid, with inhibition rates of 55.08%, 43.18%, and 41.31%, respectively. Although we did not find reports on the inhibitory effect of DESs containing glucose on α-glucosidase, there have been reports of the inhibitory effect of DESs containing other monosaccharides like fructose on α-glucosidase [28]. We speculate that since monosaccharides are products of α-glucosidase, product inhibition is highly plausible [29]. DESs with lower inhibition rates include Bet-LA, ChCl-Urea and Pro-Ethy. Water and ethanol do not have clear inhibitory activity on α-glucosidase. Therefore, when using DESs as reagents to extract α-glucosidase inhibitors, it is necessary to detect the enzyme inhibition or activation activity of the solvent itself.

In this study, DESs based on acetylcholine and malic acid, choline chloride and 1,4-butanediol, betaine and ethylene glycol, and L-proline and lactic acid had higher inhibition rates on α-glucosidase than the DES plus the extract, indicating that the DES and the extract may have antagonistic effects. This may be due to the selectivity of specific DESs in the extraction of inhibitors or other impurities that could influence the inhibitory effect. This was partially confirmed in the subsequent experiments, where the extraction efficiency of the inhibitor decreased as the solid-to-liquid ratio increased. Considering the rather clear inhibition of the extract of ChCl-Urea on α-glucosidase, the low inhibition rate of pure ChCl-Urea, and the low content of alkaloids, we chose this for further research.

### 3.2. Optimization of the Extraction Process

#### 3.2.1. Influence of Water Content

**Figure 2:**
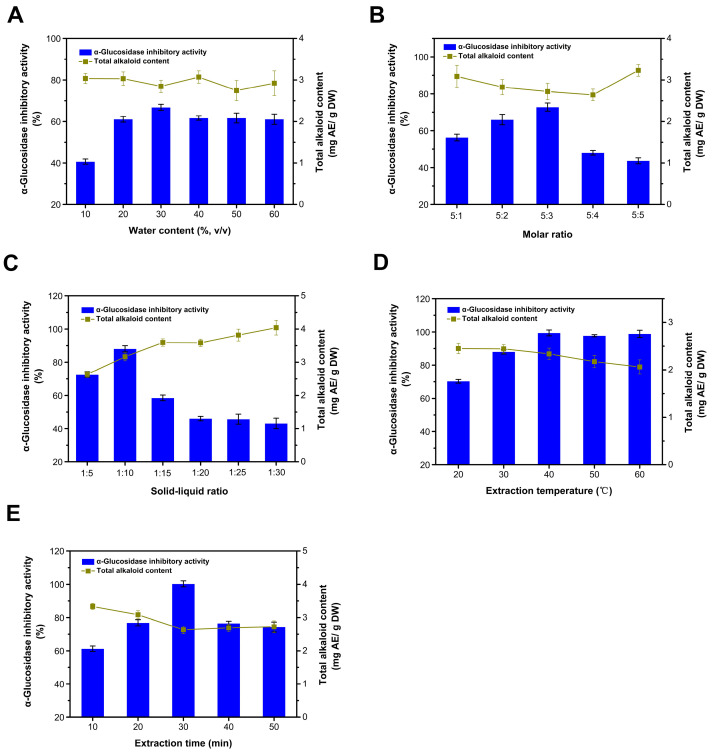
Optimization of the extraction process. Influence of water content (**A**); influence of choline chloride to urea ratio (**B**); influence of the solid to liquid ratio (**C**); influence of the extraction temperature (**D**); and influence of the extraction time (**E**).

The viscosity of DESs is mainly influenced by their composition, the molar ratio of substances, and concentration. High viscosity hinders the mass transfer of bioactive components from plant cells to the DES medium, which is a major drawback in the application of DESs for the efficient extraction of bioactive components. The water content evaluated ranged from 10 to 60% (Figure 2A). As the water content increased, the extraction efficiency of inhibitors was notably enhanced when the water content remained under 30%. However, once the water content surpassed 30%, the extraction rates of inhibitors and the alkaloids remained relatively unchanged. Under conditions of lower water content (<50% mole fraction), water can reduce the system’s viscosity and surface tension, thereby improving the extraction rate of plant bioactive components [30,31]. The DES system resists hydration by retaining its initial structure. Therefore, the increase in extraction efficiency is primarily attributed to a reduction in the viscosity of the solution [32]. However, the excessive water content in the DES solution was counterproductive to the increase in the extraction yields of target compounds, likely due to the interactions between the DES and the target compounds being weakened and the increased polarity of the mixture [30]. When the water content exceeds 43% (*w*/*w*), this results in the freeing of constituents of the DES [33,34]. Given that water itself possesses a certain level of extraction efficiency, the extraction’s inhibition rate does not significantly change with further increases in the water content (Figure 1D). Meanwhile, the water content had no significant effect on the extraction of alkaloids. Therefore, the most suitable water content was 30%.

#### 3.2.2. Influence of Choline Chloride to Urea Ratio

The molar ratio of choline chloride to urea significantly influences the yield of the inhibitor (Figure 2B). As this ratio increases, the yield of the inhibitor initially rises. Notably, the yield decreases substantially when the molar ratio exceeds 5:3. We tested two separate components, choline chloride and urea, and found that they exhibited no significant inhibitory activity on the enzyme under the tested conditions. This phenomenon could be attributed to an overabundance of hydrogen bond donors, which dilute the concentration of choline chloride in DESs, consequently affecting the interaction between the target compounds and DESs [35]. The extraction rate of alkaloids follows an inverse trend, initially decreasing and then increasing. Taking into account both the yield of the inhibitor and the alkaloid content, a molar ratio of 5:3 was deemed most suitable.

#### 3.2.3. Influence of the Solid-to-Liquid Ratio

When the solid-to-liquid ratio is maintained at or below 1:10, the yield of the inhibitor progressively increases with an increase in the solvent volume (Figure 2D). The solvent may have extracted the maximum number of extractable inhibitors at a ratio of 1:10. After this point, there was an increased dissolution of impurities, which adversely affected the enzyme’s inhibition rate [36]. As the solvent content increases, the dissolution of impurities such as alkaloids in the extraction system also increases, demonstrating the solvent’s selective affinity for the inhibitor by preferentially dissolving it first. Therefore, a solid-to-liquid ratio of 1:10 was deemed optimal, balancing higher extraction efficiency with a lower alkaloid content.

#### 3.2.4. Influence of the Extraction Temperature

The yield of the inhibitor initially increases as the extraction temperature rises, reaching its peak at 40 °C (Figure 2C). Beyond this temperature, the yield remains unchanged. This suggests that, within a certain range, elevating the temperature reduces the viscosity of DES and facilitates the extraction of the inhibitor; however, excessively high temperatures may lead to increased energy consumption [37]. Additionally, the concentration of alkaloids declines more rapidly at higher temperatures, indicating that the alkaloid’s thermal stability is inferior to that of the inhibitor. During processing, betel nuts undergo thermal treatments such as steam explosion and cooking, which leave behind active substances with substantial thermal stability [3]. Taking these observations into account, an optimal extraction temperature of 40 °C is recommended.

#### 3.2.5. Influence of the Extraction Time

Within the initial 30 min of ultrasonic treatment, the yield of the inhibitor increases (Figure 2E). When the ultrasonic time exceeds 30 min, the yield of the inhibitor declines to some extent. This suggests that ultrasound aids in the extraction of inhibitors, possibly due to its role in promoting cell lysis. Nonetheless, extended ultrasonic exposure may cause an overflow of impurities, resulting in a reduced inhibition rate. This could be attributed to the loosened tissue structure of the betel nut waste seeds after undergoing processes such as steam explosion and enzymolysis, thereby enhancing their extraction efficiency [3]. As the ultrasonic time extends, the yield of alkaloids decreases, which can be ascribed to its instability. Therefore, an optimal ultrasonic time of 30 min is recommended.

In conclusion, the optimal extraction conditions identified in this study include a moisture content of 30%, a molar ratio of 5:3, a solid–liquid ratio of 1:10, an extraction temperature of 40 °C, and an extraction duration of 30 min. Under these conditions, the inhibition rate of the extract notably increased from 66% to nearly 100%, with no significant impact on the alkaloid content. These findings suggest that optimizing the extraction conditions can enhance the efficiency of the extraction process; however, it is difficult to further reduce the alkaloid content in the extract. At the same time, these results also suggest that the extraction of inhibitors is carried out in a selective manner, while the extraction of an alkaloid is non-selective.

### 3.3. Recovery of DESs and Inhibitors

#### 3.3.1. Recycling of DESs

**Figure 3:**
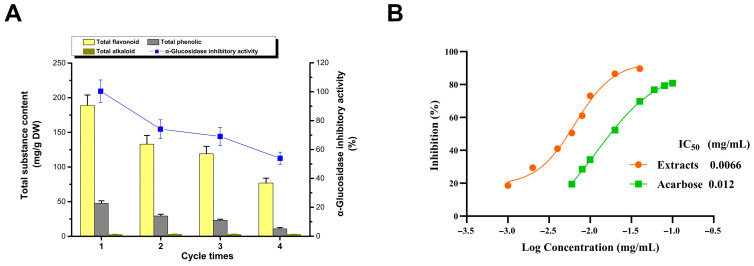
Recycling of DESs (**A**); recovery and assay of inhibitors (**B**).

The inhibitors can be recovered from the DES extract via adsorption and desorption using the macroporous resin AB-8, subsequently facilitating the reuse of DESs (Figure 3A) [38]. Under optimized extraction conditions, there is a gradual decline in extraction efficiency with successive recycling, which is particularly evident for flavonoids and polyphenols, whereas the extraction rate of the alkaloid remained relatively stable. This led to a decrease in the inhibition rate of the extracts. These observations suggest that the recycling process may diminish the selectivity of the DES, potentially due to the residual impurities in the extract, which could reduce the available interaction space between the DES and its inhibitory components. Nevertheless, even after four cycles of reuse, the inhibition rate of the DES extract (53.87%) was only marginally lower than that achieved via alcohol extraction (55.78%). This reinforces the notion that DESs not only offer exceptional extraction efficiency but also boast the advantage of recyclability.

#### 3.3.2. Recovery and Assay of Inhibitors

Following the elution of adsorbed extracts from AB-8 resin with 60% ethanol and subsequent freeze-drying, the inhibitors were obtained in powdered form (Figure 3B). The IC_50_ values for the areca nut seed extract and acarbose, the positive control, were 0.0066 and 0.012 mg/mL, respectively. This demonstrates that the extract’s inhibitory activity is significantly higher than that of acarbose. This suggests a slight reduction in the α-glucosidase inhibitory effect of processed areca nut seeds compared to fresh, unprocessed seeds [6]. Considering the relatively low alkaloid content in the waste seeds and their low material cost compared with fresh seeds, this highlights their potential for their development into antihyperglycemic agents [6]. Research on the separation and identification of effective components of α-glucosidase inhibitors is currently in progress.

## 4. Conclusions

To improve the resource utilization of betel nut waste seeds, this study assessed the impact of various solvents on the extraction of total flavonoids, polyphenols, alkaloids, and α-glucosidase inhibitors from these seeds. The investigation revealed marked disparities in both extraction efficiency and selectivity across different DESs. Importantly, certain pure DES formulations were found to inhibit α-glucosidase activity. Among these, the DES comprising choline chloride and urea was identified as superior in terms of extraction efficiency and selectivity. Consequently, the extraction conditions were optimized as follows: 30% water content, a molar ratio of 5:3, a solid–liquid ratio of 1:10, an extraction temperature of 40 °C, and a duration of 30 min. Under these conditions, the inhibition rate of the extract increased from 66% to nearly 100%. Further experimentation on the recyclability of DESs demonstrated its effective reuse for up to four batches. Moreover, the inhibitory potency of the extract, following separation and purification with macroporous resin AB-8, was significantly higher than that of acarbose. These findings underscore the advantages of DESs, including their high efficiency, selectivity, and recyclability, providing a potential direction for the high-value utilization of betel nut waste seeds.

## Figures and Tables

**Table 1:** DESs used in this study.

Solvent Abbreviation ^a^	Hydrogen Bond Acceptor	Hydrogen Bond Donor	Molar Ratio
ChCl-MA	choline chloride	malic acid	1:1
ChCl-Glu	choline chloride	glucose	5:2
ChCl-Xyl	choline chloride	xylitol	1:1
ChCl-LA	choline chloride	lactic acid	1:2
ChCl-Urea	choline chloride	urea	5:2
ChCl-Gly	choline chloride	glycerol	1:2
ChCl-But	choline chloride	1,4-butanediol	1:2
Bet-Gly	betaine	glycerol	1:1
Bet-CA	betaine	citric acid	1:1
Bet-LA	betaine	lactic acid	1:2
Pro-Gly	L-proline	glycerol	1:2
Pro-Ethy	L-proline	ethylene glycol	1:2
Pro-LA	L-proline	lactic acid	1:2

^a^ Abbreviation: ChCl, choline chloride; Bet, betaine; Pro, L-proline; MA, malic acid; Glu, glucose; Xyl, xylitol; LA, lactic acid; Gly, glycerol; But, 1,4-butanediol; CA, citric acid; Ethy, ethylene glycol.

## Data Availability

The original contributions presented in the study are included in the article, further inquiries can be directed to the corresponding authors.
